# Differential Outcomes following Optimization of Simian-Human Immunodeficiency Viruses from Clades AE, B, and C

**DOI:** 10.1128/JVI.01860-19

**Published:** 2020-05-04

**Authors:** Lawrence J. Tartaglia, Siddhant Gupte, Kevin C. Pastores, Sebastien Trott, Peter Abbink, Noe B. Mercado, Zhenfeng Li, Po-Ting Liu, Erica N. Borducchi, Abishek Chandrashekar, Esther Apraku Bondzie, Venous Hamza, Nicole Kordana, Shant Mahrokhian, Christy L. Lavine, Michael S. Seaman, Hui Li, George M. Shaw, Dan H. Barouch

**Affiliations:** a Center for Virology and Vaccine Research, Beth Israel Deaconess Medical Center, Harvard Medical School, Boston, Massachusetts, USA; b Department of Medicine, University of Pennsylvania, Philadelphia, Pennsylvania, USA; c Ragon Institute of MGH, MIT, and Harvard, Cambridge, Massachusetts, USA; Emory University

**Keywords:** SHIV, optimization, HIV-1, Env

## Abstract

Simian-human immunodeficiency virus (SHIV) infection of rhesus monkeys is an important preclinical model for human immunodeficiency virus type 1 (HIV-1) vaccines, therapeutics, and cure strategies. SHIVs have been optimized by incorporating HIV-1 Env residue 375 mutations that mimic the bulky or hydrophobic residues typically found in simian immunodeficiency virus (SIV) Env to improve rhesus CD4 binding. We applied this strategy to three SHIV challenge stocks (SHIV-SF162p3, SHIV-AE16, and SHIV-325c) and observed three distinct outcomes. We constructed six Env375 variants (M, H, W, Y, F, and S) for each SHIV, and we performed a pool competition study in rhesus monkeys to define the optimal variant for each SHIV prior to generating large-scale challenge stocks. We identified SHIV-SF162p3S/wild type, SHIV-AE16W, and SHIV-325cH as the optimal variants. SHIV-SF162p3S could not be improved, as it already contained the optimal Env375 residue. SHIV-AE16W exhibited a similar replicative capacity to the parental SHIV-AE16 stock. In contrast, SHIV-325cH demonstrated a 2.6-log higher peak and 1.6-log higher setpoint viral loads than the parental SHIV-325c stock. These data demonstrate the diversity of potential outcomes following Env375 modification in SHIVs. Moreover, the clade C SHIV-325cH challenge stock may prove useful for evaluating prophylactic or therapeutic interventions against clade C HIV-1.

**IMPORTANCE** We sought to enhance the infectivity of three SHIV stocks by optimization of a key residue in human immunodeficiency virus type 1 (HIV-1) Env (Env375). We developed the following three new simian-human immunodeficiency virus (SHIV) stocks: SHIV-SF162p3S/wild type, SHIV-AE16W, and SHIV-325cH. SHIV-SF162p3S could not be optimized, SHIV-AE16W proved comparable to the parental virus, and SHIV-325cH demonstrated markedly enhanced replicative capacity compared with the parental virus.

## Abstract (precis)

We sought to enhance the infectivity of three SHIV stocks by optimization of a key residue in human immunodeficiency virus type 1 (HIV-1) Env (Env375). We developed the following three new simian-human immunodeficiency virus (SHIV) stocks: SHIV-SF162p3S/wild type, SHIV-AE16W, and SHIV-325cH. SHIV-SF162p3S could not be optimized, SHIV-AE16W proved comparable to the parental virus, and SHIV-325cH demonstrated markedly enhanced replicative capacity compared with the parental virus.

## INTRODUCTION

Simian immunodeficiency virus (SHIV) infection of rhesus monkeys has been an important animal model for studying preventative and therapeutic strategies for human immunodeficiency virus type 1 (HIV-1). SHIVs have been utilized for the preclinical evaluation of candidate vaccines, broadly neutralizing monoclonal antibodies, and other interventions for HIV-1. SHIVs are generally constructed with a simian immunodeficiency virus (SIV) backbone and HIV-1 *tat*, *rev*, *vpu*, *and env* (1). This approach has led to the development of SHIVs from multiple HIV-1 clades (2). For example, SHIV-SF162p3 is a clade B virus that is R5-tropic and capable of replicating in memory CD4^+^ T cells (3, 4), and SHIV-1157ipd3N4 is a pathogenic clade C virus (5, 6).

The pathogenicity of SHIVs has traditionally been augmented by serial passaging in rhesus monkeys (7). Recently, Shaw and colleagues described a new strategy to produce SHIVs with improved binding to rhesus CD4 and increased replication *in vivo* (8). The phenylalanine at position 43 (F43) of CD4 engages position 375 in Env in the gp120 binding pocket (9, 10). Env375 is, thus, a critical component of the binding pocket that aids in stabilization of the CD4-Env-bound conformations during viral entry. Furthermore, sequence analyses between SIV and HIV-1 Env revealed that the naturally occurring residues in SIV at Env375 are bulky and/or hydrophobic residues such as M, H, W, Y, and F, whereas HIV-1 Env375 typically has an S residue (11). Shaw and colleagues showed that mutating Env375 to the naturally occurring amino acids found in SIV Env at this position (M, H, W, Y, and F) resulted in SHIVs with a higher *in vivo* replicative capacity (8).

Here, we applied this optimization strategy to SHIV-SF162p3 (clade B), SHIV-AE16 (clade AE), and SHIV-325c (clade C) challenge stocks (12–14). We introduced hydrophobic and/or bulky amino acid mutations into Env375 (11), and we generated 6 variants for each SHIV. We performed a pool competition study to determine the optimal variant for each SHIV, and we observed the following three distinct outcomes with this optimization procedure: SHIV-SF162p3S could not be improved, SHIV-AE16W was comparable to the parental virus, and SHIV-325cH showed greatly enhanced replicative capacity compared with the parental virus.

## RESULTS

### Generation of SHIV Env375 variants.

**FIG 1:**
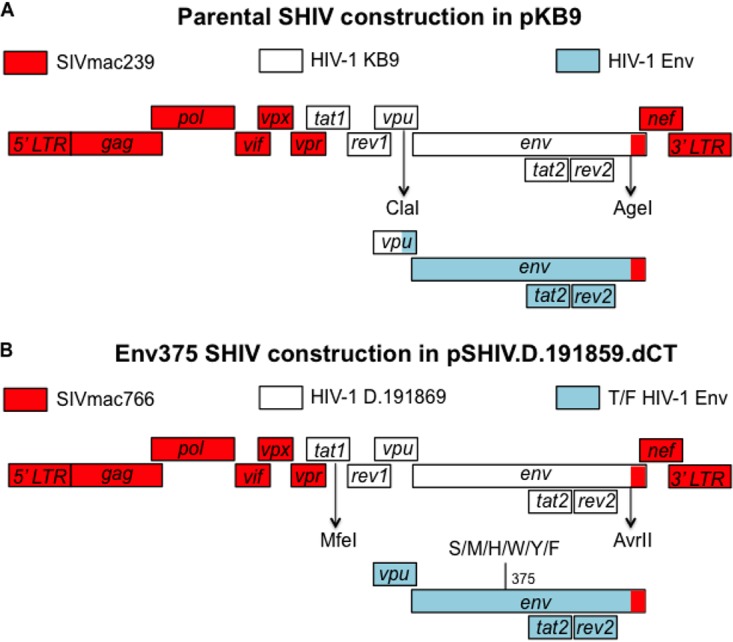
Cloning strategies comparing parental SHIVs and Env375 variant SHIVs. (A) Parental SHIVs were generated in a SIVmac239 backbone with two restriction sites, namely, ClaI and AgeI, for cloning HIV-1 Envs. (B) Env375 variant SHIVs were generated in a pSHIV.D.191859.dCT backbone with two restriction sites, namely, MfeI and AvrII, for cloning HIV-1 Envs. Site-directed mutagenesis was utilized to mutate Env375 residues.

Our lab has previously generated SHIV-SF162p3 (12), SHIV-AE16 (13), and SHIV325c (14) challenge viruses. These SHIVs were constructed using the conventional KB9 SHIV design strategy (Fig. 1A). Here, we designed a new panel of SHIVs constructed with HIV-1 *env* sequences from SHIV-SF162p3, SHIV-AE16, and SHIV325c and cloned them into a replication-competent, pathogenic SIVmac766-based SHIV.D.191859.dCT clone (Fig. 1B) (8). This clone was previously shown to have a higher replicative capacity *in vivo* than KB9-derived viruses (8). The *vpu* and *env* genes were exchanged for the corresponding regions in SHIV.D.191859.dCT.

Site-directed mutagenesis was used to substitute the wild-type amino acid at Env375 to mimic the larger and/or hydrophobic amino acids at Env375 in SIV. Env375 sequences were modified from S/wild type to M, H, W, Y, and F for SHIV-SF162p3 and SHIV-325c and from H/wild type to S, M, Y, W, and F for SHIV-AE16. A total of 6 variants for each of the three SHIVs were used for transfection in 293T cells to generate viruses. 293T cell cultures were then used to propagate each virus in either rhesus or human peripheral blood mononuclear cells (PBMCs) (Table 1). These values were established for the virus stocks after 12 to 15 days in culture with PBMCs. As we have previously reported (13, 15, 16), SHIV-SF162p3 replicated well in both rhesus and human PBMCs, whereas SHIV-AE16 and SHIV-325c replicated more efficiently in human PBMCs (Table 1). These data are consistent with growth characteristics of the parental viruses constructed in the KB9 backbone (13–15). Interestingly, the new Env375 S/wild-type, M, and H variants for SHIV-SF162p3 showed 1- to 2-log greater replication *in vitro* than the parental SHIV-SF162p3 stock (Table 1). For SHIV-AE16, all Env375 variants displayed 1- to 3-log greater replication than the parental stock. For SHIV-325c, all Env375 variants exhibited 2- to 3-log greater replicative capacity than the parental stock, and the S/wild-type, M, H, and W variants were the most replicative *in vitro*. Taken together, these findings suggest that the SHIV.D.191859.dCT backbone and the Env375 substitutions enhanced *in vitro* growth kinetics for SHIV-SF162p3, SHIV-AE16, and SHIV-325c compared with the parental viruses constructed in the KB9 backbone.

**TABLE 1:** Summary of small-scale SHIV 375 Env challenge stocks

SHIV variant by stock	PBMC type^*a*^	Viral load (RNA copies/ml)	SIV p27 levels in PBMC (ng/ml)	Infectivity titer (TCID_50_/ml)^*b*^
162p3				
Parental	Rhesus	3.48 × 10^8^	14.51	1.75 × 10^6^
S/WT^*c*^	Rhesus	1.07 × 10^9^	35.65	1.67 × 10^8^
M	Rhesus	6.24 × 10^8^	23.05	1.95 × 10^7^
H	Rhesus	1.00 × 10^9^	22.18	1.95 × 10^7^
W	Rhesus	5.89 × 10^8^	18.82	1.83 × 10^4^
Y	Rhesus	7.33 × 10^8^	20.01	7.81 × 10^5^
F	Rhesus	8.53 × 10^8^	37.63	3.91 × 10^6^
AE16				
Parental	Human	2.10 × 10^9^	102.90	1.00 × 10^4^
H/WT	Human	6.13 × 10^9^	362.20	1.95 × 10^7^
M	Human	9.43 × 10^8^	32.70	3.49 × 10^5^
S	Human	1.76 × 10^9^	45.10	3.13 × 10^4^
W	Human	6.97 × 10^8^	36.80	4.57 × 10^5^
Y	Human	2.84 × 10^9^	114.95	3.91 × 10^6^
F	Human	8.33 × 10^8^	30.25	1.75 × 10^6^
325c				
Parental	Human	2.19 × 10^9^	91.67	4.57 × 10^5^
S/WT	Human	3.76 × 10^9^	45.06	4.88 × 10^8^
M	Human	2.38 × 10^9^	67.32	>4.88 × 10^8^
H	Human	3.74 × 10^9^	93.05	>4.88 × 10^8^
W	Human	3.19 × 10^9^	78.23	>4.88 × 10^8^
Y	Human	3.78 × 10^9^	37.4	5.71 × 10^7^
F	Human	3.19 × 10^9^	49.52	5.71 × 10^7^

a PBMC type utilized for SHIV generation.

b Infectivity in TZM.bl cells (TCID_50_/ml). These values were established for the virus stocks after 12–15 days in culture with PBMCs.

c WT, wild type.

### Env375 variant pool infection study in rhesus monkeys.

**FIG 2:**
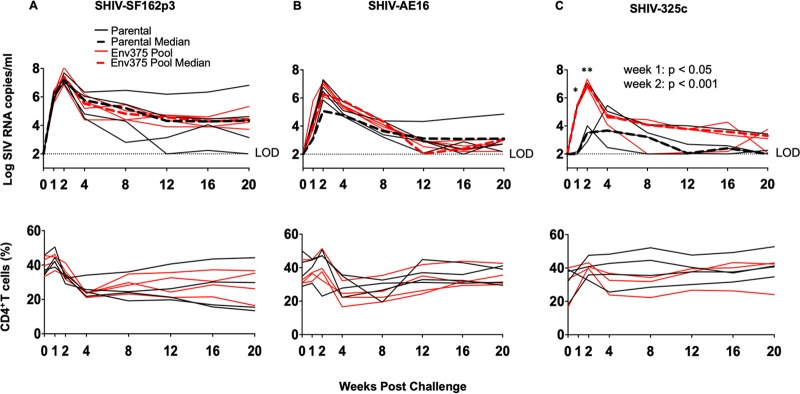
Plasma viral loads and CD4^+^ T cell counts in rhesus monkeys challenged with original or Env375 pool variants of SHIV-SF162p3, SHIV-AE16, and SHIV-325c. Plasma viral RNA (log RNA copies/ml) are shown for animals that were challenged intravenously with either the parental challenge stocks of SHIV-SF162p3 (A), SHIV-AE16 (B), or SHIV-325c (C) (black lines) or the Env375 pool variants (red line). Dashed lines indicate medians. Percentage of CD4^+^ T cell numbers in peripheral blood are shown. The dotted line reflected the limit of detection of the assay (100 RNA copies/ml). ***, *P* < 0.001; *, *P* < 0.05; unpaired *t* test (parental versus Env375 pool).

We performed a pooled competition experiment *in vivo* to define the Env375 variant from each SHIV strain with the most robust replication in rhesus monkeys. Three SHIV variant pools were constructed with Env375 variants from SHIV-SF162p3 (S/wild type, M, H, W, Y, and F), SHIV-AE16 (S, M, H/wild type, W, Y, and F), and SHIV-325c (S/wild type, M, H, W, Y, and F). Twelve adult rhesus monkeys (*n *= 4/group) were inoculated with a single intravenous (i.v.) equimolar inoculum of 10^8^ RNA copies of each SHIV pool. In this case, we used viral load rather than 50% tissue culture infective dose (TCID_50_) measurements to create pools since the *in vitro* infectivity to *in vivo* infectivity relationship was not clear since some stocks that did not grow in rhesus PMBCs grew well in rhesus monkeys. An additional 11 adult rhesus monkeys were challenged with the parental virus stocks derived from the KB9 backbone for comparison. Animals inoculated with the parental SHIV-SF162p3 stock exhibited peak viral loads ranging from 6.9- to 7.9-log RNA copies/ml at 2 weeks postinoculation, but only 3 of 4 animals had detectable viremia by week 20 (Fig. 2A). Animals that received the SHIV-SF162p3 Env375 variants had comparable peak viral loads to the parental stock at 2 weeks postinoculation, but all animals (parental and Env327 variants) maintained detectable viremia at week 20 (Fig. 2A).

Monkeys that received the parental SHIV-AE16 stock displayed peak viral loads ranging from 5.1- to 7.3-log RNA copies/ml at 2 weeks postinoculation but only showed detectable viremia in 3 of 4 animals at week 20 (Fig. 2B). Animals that received the SHIV-AE16 Env375 variants displayed peak viral loads ranging from 6.5- to 7.2-log RNA copies/ml at 2 weeks postinoculation and detectable viremia in 3 of 4 animals at week 20 (Fig. 2B).

Animals that received the parental SHIV-325c stock displayed low peak viral loads, as we previously reported, ranging from 3.7- to 5.5-log RNA copies/ml and a late peak viremia at 4 weeks postinoculation, and 2 of 3 animals had low or undetectable viremia at week 20 (Fig. 2C). In contrast, animals that were infected with the SHIV-325c Env375 variants showed median viral loads that were 3.9-log RNA copies/ml higher at peak viremia and 24-fold higher at set point viremia than the controls. Furthermore, peak viral loads ranged from 6.8- to 7.3-log RNA copies/ml at week 2 (*P* < 0.001 at week 1, *P* < 0.05 at week 2, comparing variant pool versus parental stock). Moreover, all animals that received the SHIV-32c Env375 variants were still viremic at week 20 (Fig. 2C). Taken together, these findings suggest that the pooled Env375 variants of SHIV-325c replicated to substantially higher levels than the parental SHIV-325c challenge stock. In contrast, the pooled Env375 variants of SHIV-SF162p3 or SHIV-AE16 were only modestly different than the parental SHIV challenge stocks.

### Viral sequencing.

**FIG 3:**
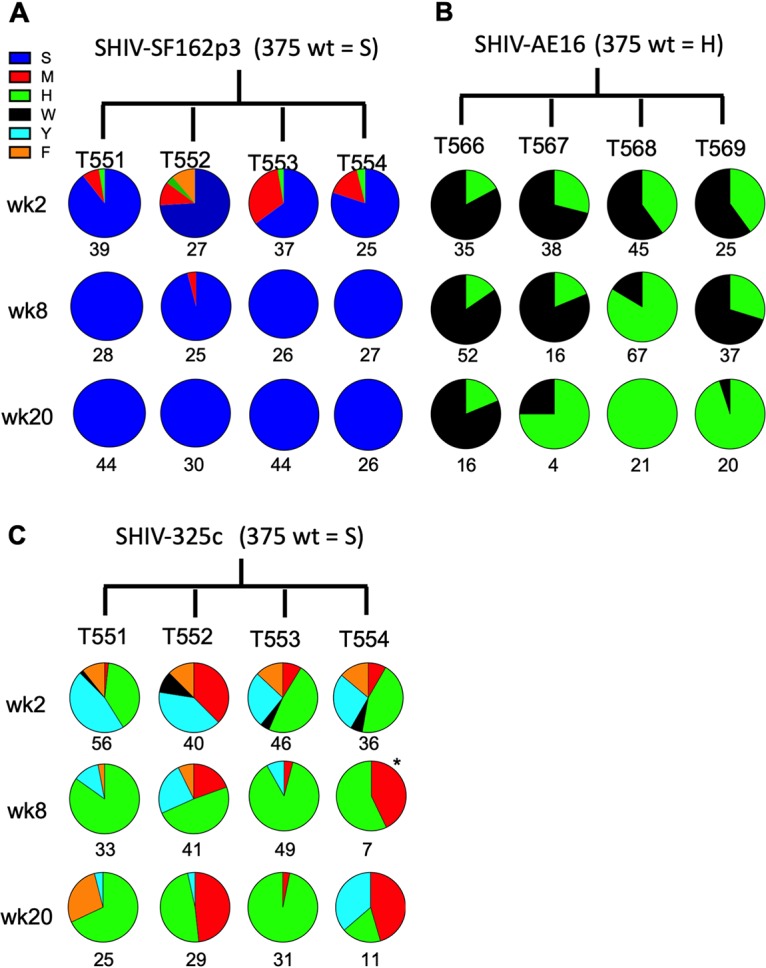
Sequence analysis of SHIV evolution in infected rhesus monkeys. Single-genome amplification was performed with plasma viral RNA on weeks 2, 8, and 20 for animals infected with SHIV-SF162p3 (A), SHIV-AE16 (B), and SHIV-325c (C) pool variants. Numbers under the circle plots indicate total sequence value per sample. Note that week 4 was assessed instead of week 8 for T562 due to low viral loads at week 8.

To define the optimal SHIV variants *in vivo*, we utilized single-genome amplification (SGA) to analyze *env* sequences from animals infected with the pooled Env375 variants for SHIV-SF162p3, SHIV-AE16, and SHIV-325c. We assessed plasma from week 2, week 8, and week 20 postinfection in each animal. In the animals infected with the SHIV-SF162p3 variants, parental Env375S was the predominant circulating variant comprising 99/128 (77%) of the total variants sequenced at week 2, with the remainder 22/128 (17%) for M, 4/128 (3%) for H, and 3/128 (2%) for F (Fig. 3A). By week 8 and 20, however, Env375S was the predominant or exclusive variant observed in 249/250 (>99%) of the total sequences (Fig. 3A). These findings indicate that the Env375S/wild-type variant of SHIV-SF162p3 had the highest replicative capacity *in vivo*. These data demonstrate that the Env375 residue in the parent SHIV-SF162p3 stock (serine) was already the optimal Env375 residue.

In the animals infected with the SHIV-AE16 variants, the Env375H from the parental stock and the Env375W variant were codominant viruses, with 45/153 (31%) positive sequences for Env375H and 78/143 (69%) for Env375W at week 2, 78/172 (45%) for Env375H and 94/172 (55%) for Env375W at week 8, and 46/82 (56%) for Env375H and 36/82 (44%) for Env375W at week 20 (Fig. 3B). Taken together, these data suggest that both the parental Env375H/wild-type and Env375W variants of SHIV-AE16 display comparable replication *in vivo*, with a slight predominance of the W variant at weeks 2 and 8 and a slight predominance of the H variant at week 20.

Lastly, in the animals infected with the SHIV-325c variants, there was a diversity of Env375 variants observed at week 2, including 23/178 (13%) for M, 60/178 (34%) for H, 64/178 (36%) for Y, 9/178 (5%) for W, and 22/178 (12%) for F. At weeks 8 and 20, the predominant Env375 variant was H at 158/226 (70%) sequences, while the remaining sequences were 33/226 (15%) for M, 24/226 (11%) for Y, and 11/226 (5%) for F (Fig. 3C). These data show that the Env375H variant of SHIV-325c had enhanced replicative capacity *in vivo* compared with the parental and other variants of SHIV-325c.

### Generation of large-scale SHIV challenge stocks.

We selected the three most prevalent Env375 variants (SHIV-SF162p3S/wild type, SHIV-AE16W, and SHIV-325cH) in the SHIV.D.191859.dCT backbone identified by SGA (Fig. 3) for the generation of large-scale challenge stocks. We inoculated 293T transfection-derived supernatant into rhesus PBMCs for SHIV-SF162p3S/wild type or into human PBMCs for SHIV-AE16W and SHIV-325cH. High titer stocks could not be generated for SHIV-AE16W or SHIV-325cH in rhesus PBMCs, despite improved replicative capacity *in vivo*. The large-scale stocks all had comparable viral load titers in the 10^9^ RNA copies/ml range, high SIV p27 levels ranging from 72.3 to 110 ng/ml, and TCID_50_/ml infectivity titers of 10^6^ for SHIV-SF162p3S/wild type and SHIV-AE16W and 10^8^ for SHIV-325cH (Table 2).

**TABLE 2:** Summary of large-scale SHIV 375 Env challenge stocks

SHIV variant	PBMC type^*a*^	Construction^*b*^	Viral load (RNA copies/ml)	SIV p27 levels in PBMC (ng/ml)	Infectivity titer (TCID_50_/ml)^*c*^
SF162p3S/WT	Rhesus	pSHIV.D.191859.dCT	1.17 × 10^9^	89.00	8.73 × 10^6^
AE16W	Human	pSHIV.D.191859.dCT	2.11 × 10^9^	72.3	9.11 × 10^6^
325cH	Human	pSHIV.D.191859.dCT	3.20 × 10^9^	110.34	>4.88 × 10^8^

a PBMC type utilized for SHIV generation.

b Plasmid backbone.

c Infectivity in TZM.bl cells.

**FIG 4:**
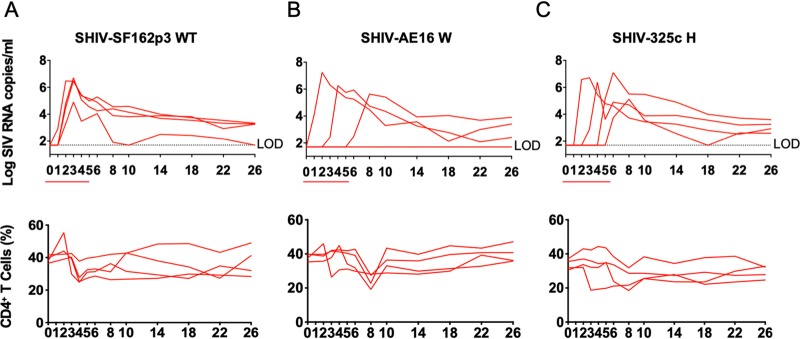
Plasma viral loads and CD4^+^ T cell counts in rhesus monkeys challenged with SHIV-SF162p3/wild-type, SHIV-AE16W, and SHIV325cH large-scale challenge stocks. Plasma viral RNA (log RNA copies/ml) are shown for animals that were challenged six times from week 0 to 5 (red line) intrarectally with 1:100-diluted SHIV-SF162p3/wild type (A), SHIV-AE16W (B), or SHIV-325cH (C). Percentage of CD4^+^ T cell numbers in peripheral blood are shown. The dotted line reflected the limit of detection of the assay (100 RNA copies/ml).

We next evaluated the infectivity of the SHIV-SF162p3S/wild-type, SHIV-AE16W, and SHIV325cH challenge stocks following repetitive intrarectal (i.r.) inoculations in rhesus monkeys. Twelve adult rhesus monkeys (*n *= 4/group) received six repetitive i.r. challenges with a 1:100 dilution of each SHIV challenge stock. For SHIV-SF162p3S/wild type, all four animals became productively infected by the second challenge, with peak viral loads ranging from 4.9- to 6.7-log RNA copies/ml and detectable viremia in 3 of 4 animals at week 26 (Fig. 4A). For SHIV-AE16W, 3 of 4 animals became infected after the first, second, and fifth challenge, respectively, with peak viral loads ranging from 5.6- to 7.3-log RNA copies/ml and with detectable viremia throughout the course of the study (Fig. 4B). For SHIV-325cH, all animals became infected after two to six challenges, with peak viral loads from 5.1- to 7.1-log RNA copies/ml, and all animals showed detectable viremia at the end of the study (Fig. 4C). These data show that the 1:100 dilutions of SHIV-SF1623S/wild-type, SHIV-AE16W, and SHIV325cH stocks infected most animals by the i.r. route and that a 1:100 dilution may be an appropriate dose for repetitive i.r. challenge regimens.

**FIG 5:**
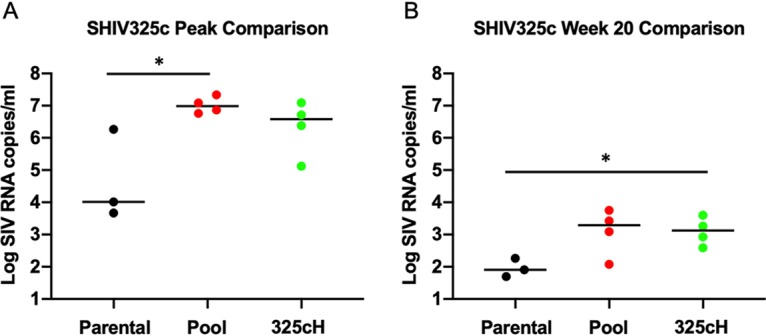
Plasma viral load comparison of SHIV-325c challenge stocks. Plasmid viral loads were analyzed for parental SHIV-325c (black), SHIV-325c pool (red), and SHIV-325cH (green) challenge stocks at peak viremia (A) and at week 20 (B). Note that week 22 plasma viral loads were used for SHIV-325cH. *, *P* < 0.05; unpaired *t* test.

We compared peak and week 20 setpoint plasma viral loads for the SHIV-325c parental, pool, and 325cH variant challenge stocks. At peak viremia, animals that received the pool and 325cH variant displayed a 2.9-log (*P* < 0.05) and 2.6-log increase, respectively, in viral loads compared with the parental stock (Fig. 5A). At week 20, animals that received the pool and 325cH variant displayed a 1.7-log and 1.6-log (*P* < 0.05) increase, respectively, compared with the parental stock (Fig. 5B). These data demonstrate that the optimized SHIV-325cH challenge stock showed markedly enhanced *in vivo* replication compared with the parental challenge stock.

### Antigenic properties of SHIV challenge stocks.

Finally, we evaluated the neutralization sensitivity of each SHIV stock in TZM-bl cells with a panel of broadly reactive neutralizing monoclonal antibodies (MAbs) as well as a panel of 5 chronic HIV+ serum samples. The SHIV-SF162p3S/wild-type, SHIV-AE16W, and SHIV-325cH challenge stocks retained neutralization profiles comparable to the parental viruses (Table 3). However, each SHIV showed higher sensitivity to neutralization by soluble human protein than the original challenge stocks, as expected for the Env375 mutations (original versus new IC_50_ values), as follows: SHIV-SF162p3S/wild type (sHuCD4 16.26 versus 12.77 μg/ml), SHIV-AE16W (sHuCD4 24.67 versus 6.08 μg/ml), and SHIV325cH (>50 versus 5.824 μg/ml). All SHIVs were fairly resistant to chronic HIV-positive serum samples and maintained a tier 2 phenotype (Table 3). Overall, these data indicate that the pSHIV.D.191859.dCT backbone as well as the Env375 amino acid substitutions did not substantially impact neutralization profiles, except for higher sensitivity to CD4 inhibition.

**TABLE 3:** Neutralization properties of SHIV-SF162p3 WT, SHIV-AE16 W, and SHIV325c H challenge stocks^*a*^

Specificity	Antibody	SF162p3 Parental	SF162p3S	AE16 Parental	AE16W	325c Parental	325cH
CD4bs	shuCD4	16.26	12.77	24.67	6.08	>50	5.824
	3BNC117	>50	0.48	>50	>50	>50	47.016
	VRC01	1.08	0.57	>50	>50	>50	43.044
	b12	2.39	2.42	>50	>50	>50	>50
	N6	0.25	0.13	1.67	2.70	9.3	8.097
V1/V2	PG9	>50	>50	0.04	0.05	1.727	1.612
	PG16	>50	>50	<0.023	<0.023	<0.023	0.039
	PGDM1400	>50	>50	<0.023	<0.023	<0.023	<0.023
	CAP256-VRC25.26	>50	>50	<0.023	<0.023	<0.023	<0.023
V3 glycan	10-1074	0.04	<0.023	>50	>50	>50	>50
	PGT121	<0.023	<0.023	>50	>50	>50	>50
	PGT128	0.37	0.10	>50	>50	>50	>50
MPER	2F5	>50	>50	36.12	13.30	>50	>50
	4E10	>50	>50	18.74	7.19	>50	>50
	10E8	12.22	3.13	1.22	1.26	44.792	22.583
HIV+ serum	HIV-018	<20	<20	<20	<20	<20	<20
	HIV-019	<20	<20	<20	<20	<20	<20
	HIV-021	<20	<20	<20	<20	<20	<20
	HIV-024	161	290	<20	<20	<20	<20
	HIV-025	<20	<20	<20	<20	<20	<20

a Assays were conducted in TZM-bl cells. Values given are neutralization titers (50% inhibitory concentrations).

## DISCUSSION

Recent reports have demonstrated that Env375 optimization can improve SHIV pathogenicity *in vivo* (8, 17). In this study, we confirm and extend these findings by describing the generation of three new SHIV challenge stocks based on SHIV-SF162p3, SHIV-AE16, and SHIV-325c. We demonstrate three distinct outcomes following Env375 optimization: (i) no improvement (SHIV-SF162p3), (ii) generation of a variant with comparable replicative capacity to the parental virus (SHIV-AE16W), and (iii) generation of a variant with improved replicative capacity compared with the parental virus (SHIV-325cH).

HIV-1 Env binding to CD4 is critical for viral entry and *in vivo* replication (8, 11, 18, 19). Li et al. showed three SHIVs (CH505, CH848, and BG505) had low baseline CD4 binding and required Env375 substitutions to enhance CD4 binding and *in vivo* infectivity (8). Our previously constructed parental SHIV-325c challenge stock (14) had a relatively low replicative capacity compared with other SHIVs. For the new variant SHIV-325cH, the Env375H substitution led to markedly enhanced replication *in vivo*. These data are consistent with previous findings that the Env375H substitution may have a selective advantage over the naturally occurring Env375S for HIV-1 clade C Envs (17).

One important aspect of our study that warrants further evaluation is to determine whether SHIV-325cH can lead to clinical AIDS progression in rhesus monkeys. Despite significant differences in viral loads measurements compared with control animals, CD4^+^ T cell counts were inconsequential. Li et al. showed in some cases that rhesus monkeys did exhibit AIDS-defining conditions after challenge (8). However, some of these animals received CD8^+^ T cell-depleting drugs that may have contributed to the high-level viremia observed. Nonetheless, these strategies suggest that further research with SHIV-325cH is warranted since clade C HIV-1 causes the majority of new HIV-1 transmissions around the world (20).

In summary, we report the generation of new SHIV-SF162p3S/wild-type, SHIV-AE16W, and SHIV-325cH challenge stocks. SHIV-325cH represents a new clade C SHIV with a high replicative capacity *in vivo* as a result of the Env375H mutation and may be useful for preclinical studies of preventative and therapeutic interventions for clade C HIV-1. This virus did not require *in vivo* passaging and was infectious in a low dose, repetitive challenge protocol in rhesus monkeys. Our data demonstrate that Env375 mutations can lead to SHIVs with reduced, comparable, or enhanced replication capacity in rhesus monkeys.

## MATERIALS AND METHODS

### Animals.

Indian-origin rhesus monkeys (Macaca mulatta) were used in the current study. Thirty-five Mamu-A*01-negative adult male and female animals were housed at Bioqual (Rockville, MD), and 12 Mamu-A*01-negative adult male and female animals were housed at Alpha Genesis (Yemassee, SC).

### Ethics.

All animals were maintained in accordance with the Association for Assessment and Accreditation of Laboratory Animals with approvals from the relevant Institutional Animal Care and Use Committees (IACUCs), including the Bioqual IACUC and the Alpha Genesis IACUC. The animal protocols in this study adhered to NIH standards set forth in the “Policy on Humane Care of Vertebrate Animals Used in Testing, Research, and Training” and the “Guidelines for the Care and Use of Laboratory Animals” (DHHS publication number NIH 85-23). Animal welfare was maintained by the Bioqual and Alpha Genesis animal management programs, which are accredited by the American Association for the Accreditation of Laboratory Animal Care (AAALAC) and meet all applicable federal and institutional standards for standard housing, standard monkey diet, water *ad libitum*, social enrichments, and steps intended to minimize suffering, such as the use of anesthesia for all procedures and analgesia for invasive procedures.

### Cells.

Human and rhesus monkey peripheral blood mononuclear cells (PBMCs) were isolated by Ficoll-Hypaque gradient purification, followed by stimulation with concanavalin (ConA; 6.25 μg/ml) and human interleukin-2 (IL-2; 20 units/ml; AIDS Reagent Program) overnight. Human whole blood was purchased commercially (Research Blood Components), and rhesus whole blood was purchased from Bioqual for PBMC isolation. Cells were cultured in RPMI 1640 medium (Gibco) supplemented with 20% fetal bovine serum, 2 mM glutamine, 100 units/ml penicillin, 100 μg/ml streptomycin, and 20 units/ml of IL-2. TZM-bl cells (JC53-bl clone 13 cells) are derived from a HeLa cell line (JC.53) that stably expresses CD4 and HIV coreceptors, as well as luciferase and β-galactosidase, under the control of the HIV-1 long terminal repeat.

### Design and construction of SHIV molecular clones.

HIV-1 envelope sequences from SHIV-SF162p3, SHIV-AE16, and SHIV-325c, previously constructed in the KB9 backbone (8, 13–15), were used to design a new panel of SHIVs in an optimized SHIV.D.191859.dCT backbone (8, 21, 22). The SHIV.D.191859.dCT backbone containing a MfeI restriction site upstream of *vpu* and an AvrII restriction site at the C-terminal end of *env* was provided by George Shaw, University of Pennsylvania, Philadelphia, PA. In order to facilitate the cloning of SHIV-SF162p3, SHIV-AE16, and SHIV325c, site-directed mutagenesis was performed with the Q5 site-directed mutagenesis kit (New England BioLabs) to introduce silent mutations into *vpu* and *env* sequences synthesized by GeneArt (GeneArt, Invitrogen, Darmstadt, Germany) to remove internal MfeI and AvrII restriction sites, leaving only MfeI and AvrII sites flanking the 5′ and 3′ ends. Bulky and/or hydrophobic amino acids were substituted for the wild-type amino acid at position 375 in the HIV *env* sequence, using primers shown in Table 4, as follows: SHIV-SF162p3 (S → M, H, W, Y, and F), SHIV-AE16 (H → S, M, W, Y, F), and SHIV-325c (S → M, H, W, Y, and F). Synthetic genes were digested and subcloned into the corresponding regions of the pSHIV.D.191859.dCT backbone using MfeI and AvrII. Plasmids containing the SHIV infectious molecular clones were transfected into 293T cells using LipoD293 (SigmaGen Laboratories, Rockville, MD). Cell culture supernatants were collected after 48 h and clarified through a 0.45-μm-pore-size filter.

**TABLE 4:** Site-directed mutagenesis primer sequences

Primer name	Primer sequence
SHIV-SF162p3	
SHIV-SF162pp3.His.Fwd	TGTAATGCACCATTTTAATTGTGGAGGGGAATTTTTC
SHVI-SF162p3.Met.Fwd	TGTAATGCACATGTTTAATTGTGGAGGGGAATTTTTC
SHIV-SF162p3.Tyr.Fwd	TGTAATGCACTATTTTAATTGTGGAGGGGAATTTTTC
SHIV-SF162p3.Trp.Fwd	TGTAATGCACTGGTTTAATTGTGGAGG
SHIV-SF162p3.Phe.Fwd	TGTAATGCACTTTTTTAATTGTGGAGGGGAATTTTTC
SHIV-SF162p3.Uni.Rev	ATTTCTGGGTCCCCTCCTG
SHIV-AE16	
SHIV-AE16.Ser.Fwd	TACAACGCATAGTTTTAATTGTAGAGGG
SHIV-AE16.Met.Fwd	TACAACGCATATGTTTAATTGTAGAGGGGAATTTTTC
SHIV-AE16.Tyr.Fwd	TACAACGCATTATTTTAATTGTAGAG
SHIVAE-16.Trp.Fwd	TACAACGCATTGGTTTAATTGTAGAGGGG
SHIVAE-16.Phe.Fwd	TACAACGCATTTTTTTAATTGTAGAGGG
SHIVAE-16.Uni.Rev	ATTTCTAGATCTCCTCCTG
SHIV-325c	
SHIV-325c.His.Fwd	TACAACACATCACTTTAATTGTAGAGGAG
SHIV-325c.Met.Fwd	TACAACACATATGTTTAATTGTAGAGGAGAATTTTTC
SHIV-325c.Tyr.Fwd	TACAACACATTACTTTAATTGTAGAGGAG
SHIV-325c.Trp.Fwd	TACAACACATTGGTTTAATTGTAGAGG
SHIV-325c.Phe.Fwd	TACAACACATTTCTTTAATTGTAGAGGAG
SHIV-325c.Uni.Rev	ATTTCTAAGTCCCCTCCTG

### Generation of SHIV challenge stocks.

Six infectious molecular clones of SHIV-SF162p3S/wild type, M, H, Y, W, and F replicated to high levels in rhesus PBMCs, whereas six infectious molecular clones of SHIV-AE16S, M, H/wild type, W, Y, and F and six infectious molecular clones of SHIV-325cS/wild type, M, H, W, Y, and F replicated to high levels in human PBMCs. SHIV-SF162p3S/wild type, SHIV-AE16W, and SHIV-325cH were selected to generate large-scale challenge stocks. Each virus stock was produced from 120 ml of rhesus monkey or human whole blood. Cell culture supernatants harvested from transiently transfected 293T cell were used to inoculate ConA-stimulated PBMCs in the presence of 20 U/ml human IL-2 (AIDS Research and Reference Reagent Program). The medium was replaced and collected in 3-day increments over the course of 12 to 15 days. Virus was quantified by SIV p27 enzyme-linked immunosorbent assay (ELISA; Zeptometrix), 50% tissue culture infectious dose (TCID_50_) in TZM-bl cells, and reverse transcription-quantitative PCR (RT-qPCR).

### TCID_50_ titer in TZM-bl cells.

Virus stocks were assessed for infectivity by inoculation of TZM-bl cells using 5-fold serial dilutions in the presence of 11 μg/ml of diethylaminoethyl (DEAE)-dextran hydrochloride (Sigma, St. Louis, MO) in quadruplicate wells. Virus infectivity was determined 48 h postinfection by measuring the levels of luciferase activity expressed in infected cells. The TCID_50_ was calculated as the dilution point at which 50% of the cultures were infected.

### Neutralization assay.

Neutralization assays were performed in duplicate in TZM-bl cells, as previously described (23, 24). This assay measures a decrease in luciferase reporter gene expression following single-round viral infection of TZM.bl cells. Briefly, 5-fold serial dilutions of monoclonal antibody (MAb) reagents were performed in duplicate and incubated with SHIVs for 1 h at 37°C. TZM.bl cells were then added in growth medium containing 37°C and 5% CO_2_. Luciferase reporter gene expression was measured using a Bright-Glo luciferase reagent (Promega) and a Victor 3 luminometer (Perkin Elmer). Neutralization titers (50% inhibitory concentrations [IC_50_s]) were calculated as the sample dilution at which the relative luciferase units (RLUs) were reduced by 50% compared with RLUs in virus control wells after subtraction of background RLUs in cell control wells. Soluble human CD4 protein (Progenics) and MAbs 3BNC117 and 10–1074 (provided by Michel Nussenzweig, The Rockefeller University); VRC01, CAP256-VRC25.26, and 10E8 (provided by John Mascola, Vaccine Research Center); N6 (provided by Mark Connors, National Institutes of Health); PGDM1400 and PGT121 (Catalent); PGT128 (provided by Dennis Burton, The Scripps Research Institute), and MAbs b12, PG9, PG16, 2F5 (Polymun), and 4E10 were purchased from Polymun (Klosterneuburg, Austria). The HIV+ serum samples were obtained from chronically infected individuals (not on ART) at Brigham and Women’s Hospital (Boston, MA) under an IRB-approved blood draw protocol by Lindsey Baden (Brigham and Women’s Hospital, Boston, MA).

### Single-genome amplification.

SGA assays were performed as previously described (25) except that universal primers used here were designed to amplify conserved regions of SHIV-SF162p3, SHIV-AE16, and SHIV-325c. Briefly, viral RNA was isolated and reverse transcribed to viral cDNA using SHIV.SGA.Uni.RT.Rev.2 (5′-TGAATACAGAGCGAAATGCAGTG-3′). First-round outer PCR was performed with Platinum PCR SuperMix high fidelity mix (ThermoFisher Scientific) together with primers SHIV.SGA.Uni.Fwd.2 (5′-ACTCTTCATGCATTTCAGAG-3′) and SHIV.SGA.Uni.Rev.1 (5′-ACTAATTTCCATAGCCAGC-3′). PCR conditions were programmed as follows: 1 cycle of 94°C for 30 sec; 35 cycles of 94°C for 30 sec, 55°C for 30 sec, and 68°C for 4 min; followed by a final extension of 68°C for 5 min. Approximately 1.5 μl of first-round PCR product was added to the Platinum PCR SuperMix with primers SHIV.SGA.Uni.Fwd.2 (5′-ACTCTTCATGCATTTCAGAG-3′) and SHIV.SGA.Uni.RT.Rev.2 (5′-TGAATACAGAGCGAAATGCAGTG-3′). PCR parameters were programmed the same as above but increased to 45 cycles for second-round PCR. Amplicons from cDNA dilutions resulting in ∼30% positive were processed for sequencing.

### RT-qPCR.

RT-PCR assays were utilized to monitor viral loads, essentially as previously described (26). RNA was extracted from plasma with a QIAcube high-throughput (HT) instrument (Qiagen, Germany) using the QIAcube 96 Cador pathogen HT. The SIV Gag gene was utilized as a standard. RNA standards were generated using the AmpliCap-Max T7 high-yield message maker kit (Cell Script) and purified with RNA clean and concentrator kit (Zymo Research, CA, USA). RNA quality and concentration were assessed by the Beth Israel Deaconess Medical Center (BIDMC) Molecular Core Facility. Log dilutions of the RNA was included with each RT-qPCR assay. Reverse transcription of both standards and samples was done using the Superscript III VILO kit (Invitrogen). RT-PCR was run on the Quantstudio 6 Flex system (Applied Biosystems). Viral loads were calculated as virus particles (VPs) per ml. Assay sensitivity was >100 copies/ml.

### Intravenous inoculation of SHIV variants.

Animals received a single intravenous inoculation of the Env375 pool variants of SHIV-SF162p3 (S/wild type, M, H, W, Y, and F), SHIV-AE16 (S, M, H/wild type, W, Y, and F), or SHIV-325c (S/wild type, M, H, W, Y, and F) challenge stocks. Additional groups received a single intravenous inoculation of the parental SHIV-SF162p3, SHIV-AE16, or SHIV-325c challenge stocks for comparison. Animals were monitored for viremia and CD4^+^ T cell depletion by analyzing blood plasma samples via viral load assays and CD4^+^ T cell counts, respectively.

### Challenge study.

Four animals per SHIV group received six repetitive intrarectal (i.r.) inoculations of 1 ml of 1:100-diluted SHIV-SF162p3S/wild-type, SHIV-AE16W, and SHIV-325cH challenge stocks. All animals were monitored for viral loads and CD4^+^ T cell counts.
